# Satisfaction with maternal health services and associated factors among parents in Ethiopia: a systematic review and meta-analysis

**DOI:** 10.3389/fpubh.2025.1401498

**Published:** 2025-09-24

**Authors:** Agerie Mengistie Zeleke, Worku Chekol Tassew, Gashaw Melkie Bayeh, Yeshiwas Ayale Ferede

**Affiliations:** ^1^Department of Midwifery, Debark University College of Health Science, Debark Town, Ethiopia; ^2^Departments of Nursing, Tedda Health Science College, Gondar City, Ethiopia; ^3^Department of Environmental Health, College of Medicine and Health Science, Injibara University, Injibara, Ethiopia; ^4^Department of Midwifery, Tedda Health Sciences College, Gondar City, Ethiopia

**Keywords:** satisfaction, knowledge, maternal health, services, Ethiopia

## Abstract

**Introduction:**

Satisfaction with maternal health services serves as a vital indicator of the overall quality of care and significantly influences child healthcare outcomes. However, there remains a deficiency of comprehensive evidence concerning parents’ satisfaction with these vaccination services. This review aimed to evaluate the level of satisfaction with maternal health services and identify the factors that influence it among parents in Ethiopia.

**Methods:**

Satisfaction with maternal health services serves as a vital indicator of the overall quality of care and significantly influences child healthcare outcomes. However, there remains a deficiency of comprehensive evidence concerning parents’ satisfaction with these maternal health services. This review aimed to evaluate the level of satisfaction with maternal health services and identify the factors that influence it among parents in Ethiopia.

**Results:**

In this review, a total of 13 studies comprising 6,023 participants were included in the analysis. The findings indicated that the level of parental satisfaction with maternal health services was 63.5% (95% CI: 58.74, 72.44%). Several factors were identified as contributors to parental satisfaction with maternal health services, including knowledge about vaccination services (AOR = 1.93; 95% CI: 1.09, 3.42), favorable attitudes toward maternal health services (AOR = 3.23; 95% CI: 1.86, 5.62), welcoming approaches (AOR = 3.89; 95% CI: 1.80, 8.42), and the experience of waiting less than 30 min to receive services (AOR = 2.59; 95% CI: 1.25, 3.35).

**Conclusion:**

Overall, only two-thirds of parents reported satisfaction with maternal health services. Key factors influencing this level of satisfaction included the healthcare providers’ welcoming approach, waiting times of under 30 min at health facilities, and parents’ knowledge and attitudes toward vaccination. To enhance parental satisfaction, it’s essential to address these identified factors. Improving timely and welcoming interactions in healthcare service delivery is highly recommended. Ultimately, boosting parental satisfaction with maternal healthcare services calls for a comprehensive approach that emphasizes respectful interactions and efficient service delivery.

**Systematic review registration:**

PROSPERO ID CRD42024577054.

## Introduction

The World Health Organization (WHO) launched the Expanded Program on Immunization (EPI) in 1974 to address vaccine-preventable diseases (1). The EPI is recognized as one of the most significant public health initiatives globally, as it has substantially reduced morbidity and mortality related to childhood infections (2). Its effective implementation ensures high-quality care, making it a vital component of healthcare delivery systems. EPI services provide valuable insights into service operations from the clients’ perspectives and highlight the necessary adjustments to meet their expectations (3, 4).

In 2021, global immunization reports revealed that approximately 25 million children were either unvaccinated or received insufficient vaccinations in certain areas (5). Nearly 3 million children die each year from vaccine-preventable infectious diseases, with around 2 million of these deaths occurring in developing countries before the age of five (6). In sub-Saharan African nations, about 4.4 million child deaths annually are linked to inadequate immunization coverage, primarily due to communicable diseases that could be prevented through vaccination (7). Although the Ethiopian Ministry of Health’s sector transformation plan aimed to prioritize child health and achieve 100% immunization coverage for all children under two by 2021, universal childhood immunization has largely not been realized (8). This gap has led to an estimated 472,000 children dying each year before their fifth birthday, mainly from vaccine-preventable diseases (9). According to the Ethiopian Mini Demographic and Health Survey (EDHS), national immunization coverage was only 76% in 2019, falling short of the national target of 100% by 2025 (10). In comparison, an Ethiopian child is 30 times more likely to die before the age of five than a child in Western Europe (11). This concerning underreporting rate presents serious risks, including fatalities, disabilities, the resurgence of previously eradicated infectious diseases, and negative health outcomes for children.

Parental satisfaction with the services offered is a crucial aspect of healthcare quality and is closely linked to the use of maternal and child health services (12). Valuing parents’ autonomy, dignity, emotions, and preferences creates a positive psychological environment that improves clinical outcomes and client retention, while also decreasing the risk of malpractice claims related to EPI services (13). The perceived satisfaction level of parents visiting healthcare facilities serves as a key indicator of the quality of EPI services (14). To enhance parental satisfaction, various interventions have been attempted (15). Recent initiatives have aimed at preventing abusive and disrespectful maternal care to improve child immunization coverage through the effective delivery of quality care (16, 17). Satisfied parents are more inclined to return for necessary services and recommend these services to others, making satisfaction a vital measure of healthcare quality (15). Research has suggested the importance of providing client-centered, high-quality services for children under 5 years old (18). However, challenges persist in addressing parental satisfaction during child immunization services. These challenges need to be tackled through summarized evidence to enhance the quality of healthcare service delivery (19). Without addressing parental satisfaction during routine maternal health services, Ethiopia has faced difficulties in achieving its targeted figures and sustainable development goals (20). Parental satisfaction reflects how well parents believe their needs and expectations are met by the services offered, which is crucial for encouraging respectful and evidence-based care to increase child immunization rates and enhance care quality (21). Various initiatives focus on enhancing the service process, ensuring a steady supply of vaccines and logistics, and providing training on child vaccinations (22, 23). Despite providing various training programs focused on child vaccinations, significant challenges persist regarding the capacity of healthcare providers across the nation. This affects the delivery of high-quality standard services for children under 5 years old (24).

To date, there have been different (25–27) studies conducted on the level of parental satisfaction with childhood immunization in Ethiopia. However, these studies have revealed inconsistent findings on the level of satisfaction with childhood vaccines, ranging from 37.4% (28) to 84.65% (29), and varying degrees of quality scores. Furthermore, the impact of various socio-demographic, socioeconomic, varying healthcare provider approaches, and a lack of technical support on the level of parental satisfaction in Ethiopia remains inconclusive (30, 31). The main goal of this review was to summarize the findings from thirteen primary studies on the level of satisfaction with childhood vaccines. By consolidating these studies, it becomes easier to summarize their results. This was the first systematic review and meta-analysis on satisfaction with childhood vaccines and their associated factors in Ethiopia.

Therefore, this review aims to assess the pooled level of parental satisfaction with childhood vaccination services and associated factors in Ethiopia. The findings will help quality assurance activities that assess parental satisfaction with maternal health service care can provide valuable insights into children’s healthcare experiences. Client satisfaction serves as an effective proxy indicator for measuring health outcomes and plays a crucial role in enhancing the quality of health services. Improved quality of maternal health services is expected to lead to increased maternal health service coverage both in specific service areas and across the country. Furthermore, identifying factors associated with parental satisfaction regarding maternal health services is essential for encouraging adherence to maternal health services and achieving the intended control of vaccine-preventable diseases.

## Materials and methods

The Preferred Reporting Items for Systematic Reviews and Meta-Analyses (PRISMA) checklist was utilized (Supplementary Table S1, PRISMA Checklist, 2020). Systematic Review Registration: (PROSPERO ID CRD42024577054).

### Search strategy and data sources

This review and meta-analysis were developed based on the PRISMA (Preferred Reporting Items for Systematic Review and Meta-Analysis) guideline (32). Studies about the level of satisfaction toward maternal health services were identified through an online search of Scopus, Web of Science, PubMed/Medline, Science Direct, African Journal Online, and the Wiley Online Library from January 3 to 30, 2024. Additionally, a Google Scholar search was performed as a confirmatory measure to ensure that no primary studies were overlooked. The focus of this research was specifically on the level of satisfaction with maternal health services in Ethiopia. Medical Subject Headings thesaurus (MeSH) terms were searched with appropriate combinations of Boolean operators: “Satisfaction with maternal health services OR “prevalence of satisfaction with maternal health services,” OR “level of satisfaction with childhood Immunization services,” AND “parent,” OR” women,” OR “guardian,” OR “caregiver,” AND “determinant factors,” OR “associated factors” (Supplementary Table S2).

### Study inclusion and exclusion criteria

All primary studies that utilized observational study designs, specifically cross-sectional studies, to assess the level of satisfaction with maternal health services and the associated factors were included. The predefined eligibility criteria were as follows:

#### Population

The participants needed to be parents (including fathers, mothers, and guardians) utilizing maternal health services.

#### Exposure

The focus was on determinants or factors associated with the level of satisfaction regarding maternal health services.

#### Study location

The studies were required to be conducted in Ethiopia.

#### Study design

All observational studies were considered.

#### Publication status

Both published articles and gray literature were included. From these 10 articles were published and three studies were unpublished.

#### Language

Peppers searched without language restriction; however, published only articles in English were considered.

The search included all primary published articles without restrictions on the year of publication. However, the articles retrieved were from the years 2016 to 2024.

Studies that did not state outcome measures, had study populations differing from parents regarding maternal health services, or were review articles, case series, letters, comments, or editorials were excluded. The inclusion criteria were defined using a modified Condition, Context/Setting, and Population (CoCoPop) framework (see Table 1). Searched associated factors like, sociodemographic characteristic, approaches of healthcare providers, parents’ knowledge and attitudes toward vaccination were searched from each included articles.

**Table 1:** Framework for determining the eligibility of studies of the prevalence of Satisfaction with maternal health Services and associated factors among parents in Ethiopia (CoCoPop).

Mnemonics	CoCoPop (condition, contest/setting, population)			
	Conditions	Factors	Population	Context/Setting
	Satisfaction with Maternal Health Services	Determinants associated factors	Parents, Women, Father, Guardians	Ethiopia
Keywords	Satisfaction	Associated factors	Parents	Ethiopia

### Study screening and selection

First, four researchers (AMZ, YAF, WCT, and GMB) evaluated the studies using specific inclusion and exclusion criteria. They started by analyzing the titles and abstracts of the studies found in the databases. Then, eliminate duplicate research from each database that was exported to the citation manager program EndNote X 7. Afterward, the selected studies underwent a full-text screening. The PRISMA flow diagram was used to document the reasons for including or excluding each study. Finally, a list of studies eligible for data extraction in the systematic review and meta-analysis was prepared (Table 2). The data were extracted independently by three authors (AMZ, YAF, and WCT) reviewed the extracted data and engaged in discussions with the data extractors to ensure accuracy.

**Table 2:** Parents’ satisfaction toward maternal health services and associated factors in Ethiopia, 2024.

Author first name	Pub Year	Region	Study population	Sample size	Participants	Sampling technique	Prevalence	Quality score
Mohamed C, (43)	2021	Harar	Parent	407	392	SSRS	79.1	9
Zeleke M, et al. (39)	2023	Amhara	Parent	556	554	MSS	69.3	9
Yohans (40)	2020	Sidama	Parent	545	520	SSRS	55.2	9
Gebre Eyes us F, et al. (3)	2020	Amhara	Parent	682	682	MSS	68.2	9
Tadele M. (41)	2020	Oromia	Parent	497	477	SSRS	74.8	9
Tesfaye E. (33)	2023	Addis Ababa	Parent	366	366	SRS	61.2	9
Tshaye S, et al. (45)	2023	Amhara	Parent	245	244	SRS	58.9	8
Terefe. L, et al. (45)	2023	Southern Ethiopia	Parent	402	400	SSRS	47.2	9
Dona E, et al. (46)	2021	Southern Ethiopia	Parent	422	404	SSRS	76.7	8
Husen A, et al. (42)	2016	Amhara	Parent	422	419	SRS	61.1	9
Debela B, et al. (29)	2022	Amhara	Parent	722	705	MSS	84.65	9
Lafore, M (28)	2021	Southern Ethiopia	Parent	478	478	SSRS	37.4	9
Erchafo. E (44)	2016	Southern Ethiopia	Parent	382	382	SRS	66.2	9

MSS, multi-stage random sampling; SRS, simple random sampling; SSRS, systematic random sampling.

#### Outcome measurements

The study focused on assessing parents’ level of satisfaction with maternal health services. According to the included primary studies, the level of parents’ satisfaction was measured using (5-point Likert scale) questions (33–35). Factor mean was computed for each participant, and participants with a score equal to or above the factor were considered as parents were classified into two categories regarding their overall satisfaction with women’s health services: “satisfied” and “not satisfied.”

#### Quality assessment

The Newcastle-Ottawa Scale was utilized to assess the quality of the studies included in the review (36). This tool is structured into three main components. The first component consists of five stars and evaluates the methodological quality, considering factors such as the sampling process, sample size, response rate, and the identification of exposure or risk factors. The second component focuses on the comparability of the studies, with a maximum of two stars available. Lastly, the third component appraises the outcomes and statistical analyses of the primary studies, allowing for up to three stars. Lastly, the third component appraises the outcomes and statistical analyses of the primary studies, allowing for up to three stars.

Overall, this quality assessment tool contains 9 items that pertain to the risk of bias, producing scores that span from a minimum of “0” to a maximum of “9.” The risk of bias is categorized as low quality for scores under 6 and high quality for scores between 7 and 9. To ensure the reliability of the quality assessments, two authors (YAG and GMB, WCT) performed quality assurance checks, with any disagreements regarding the evaluations of the articles resolved through collaboration and discussion among all authors.

#### Publication bias and heterogeneity

We employed Cochran’s Q and I-squared statistics to evaluate heterogeneity among the studies. The interpretations for these statistics are as follows: a 0 to 40% range suggests that heterogeneity may not be significant, 40 to 60% indicates moderate heterogeneity, 60 to 90% signifies substantial heterogeneity, and a range of 90 to 100% denotes considerable heterogeneity (37). To investigate publication bias, we utilized both the funnel plot method for subjective observation and Egger’s test, where a *p*-value below 0.05 indicated the presence of publication bias (38). Additionally, a sensitivity analysis (leave-one-out) was performed to determine the impact of any individual study on the overall pooled prevalence, assuming homogeneity across groups. Subgroup analysis was also conducted based on the type of sampling technique used in the studies.

#### Data processing and analysis

Standardized data were extracted using the standard Microsoft Excel format by two authors (AMZ and WCT) and then exported into STATA version 11 software for further analysis. The following data were extracted from the included studies: name of primary author, publication year, and country’s region of the study, sample size, prevalence, study population, sampling technique, study design, and the selected predictors of the level of satisfaction with childhood vaccination services. The second author (YAF) revised the extracted data and discussed any discrepancies between the data extractors. Disagreements that occurred during data extraction were resolved through discussions among all authors. To combine the outcome data from accepted research, the authors employed the random-effects model. The data were reported as pooled outcome variables with a 95% confidence interval. Statistical heterogeneity was checked using the Cochrane Q-test and I^2^ statistics.

## Results

### Studies selection

**Figure 1:**
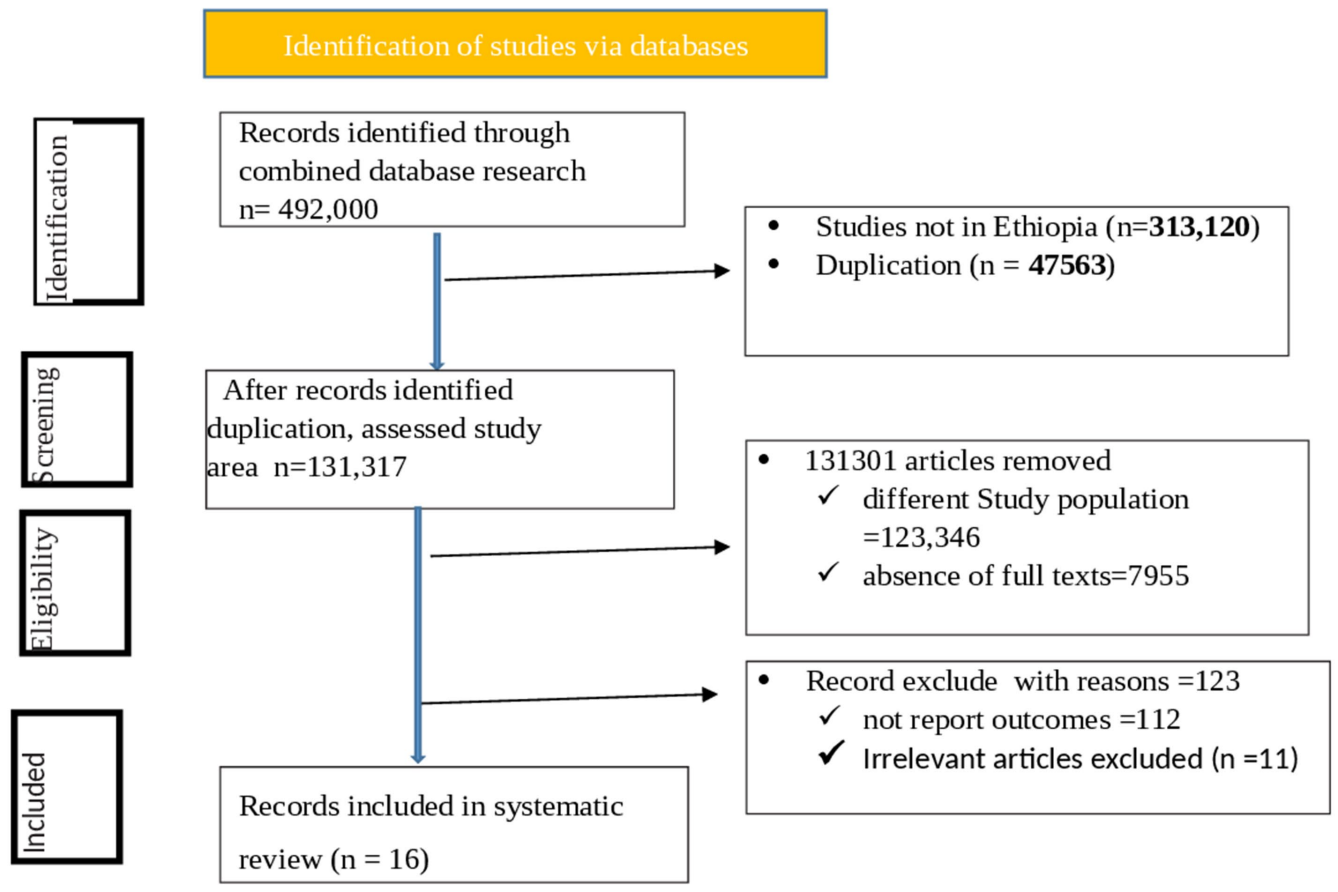
PRISMA flow chart for systematic review and meta-analysis.

The first search identified 146,987 articles from PubMed, Web of Science, Science Direct, African Journal Online, Google Scholar, and the Wiley Online Library. Of them, 97,000 articles were not studies conducted in Ethiopia, and 33,333 articles removed with different study populations, 2,683 due to duplications, and 9,955 articles excluded because of the absence of full texts. Ultimately, thirteen studies were included in the systematic review and meta-analysis, totaling 6,023 study participants (Figure 1).

### Summary of primary studies

Regarding study design, all (100%) of the studies were cross-sectional, and thirteen (100%) of the studies had high-quality articles assessed by the Newcastle Ottawa Scale (≥7). The overall level of parental satisfaction with maternal health services was reported in various regions in Ethiopia; five (38.46%) of the 13 studies were from the Amhara Regional State, and four (30.77%) of the thirteen studies were from Southern Ethiopia. The sample sizes ranged from 245 to 722 studies, with to smallest to the greatest difference in sample size (Table 2).

### The level of parents’ satisfaction with maternal health services

**Figure 2:**
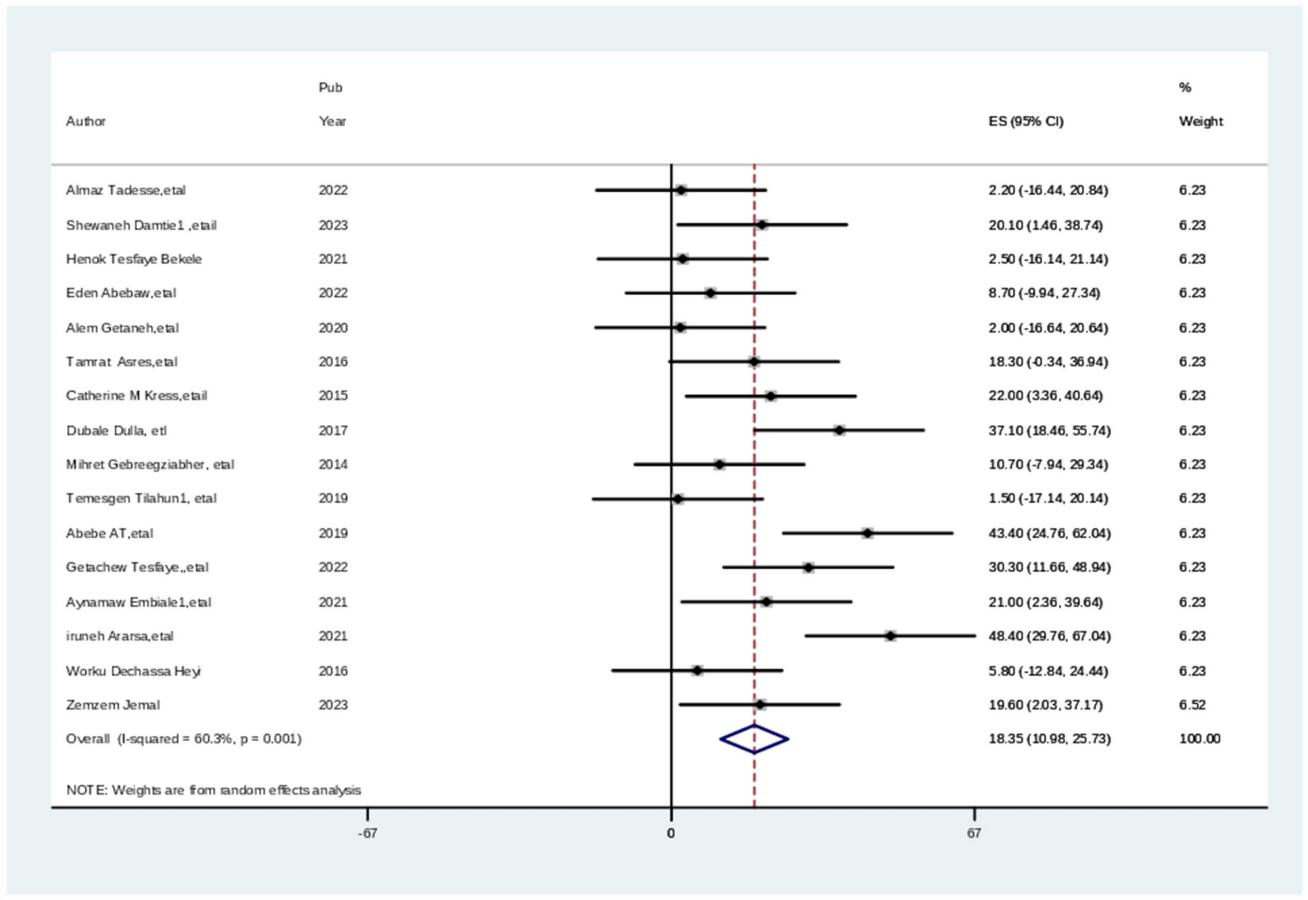
The pooled estimates the practice of healthcare professionals toward cervical cancer screening in Ethiopia.

The pooled data from 6,023 study participants were used to estimate the level of satisfaction with maternal health services using meta-analysis. The overall pooled level of satisfaction with childhood immunization services was 63.6% (95% CI: 58.74, 72.44%) (Figure 2) with substantial heterogeneity across the studies (chi^2^ = 59.81 (d.f. = 12), (*p* = 0.000), and I^2^ = 79.9%) due to differences in healthcare infrastructure, cultural attitudes, or regional policies.

### Subgroup analysis

**Figure 3:**
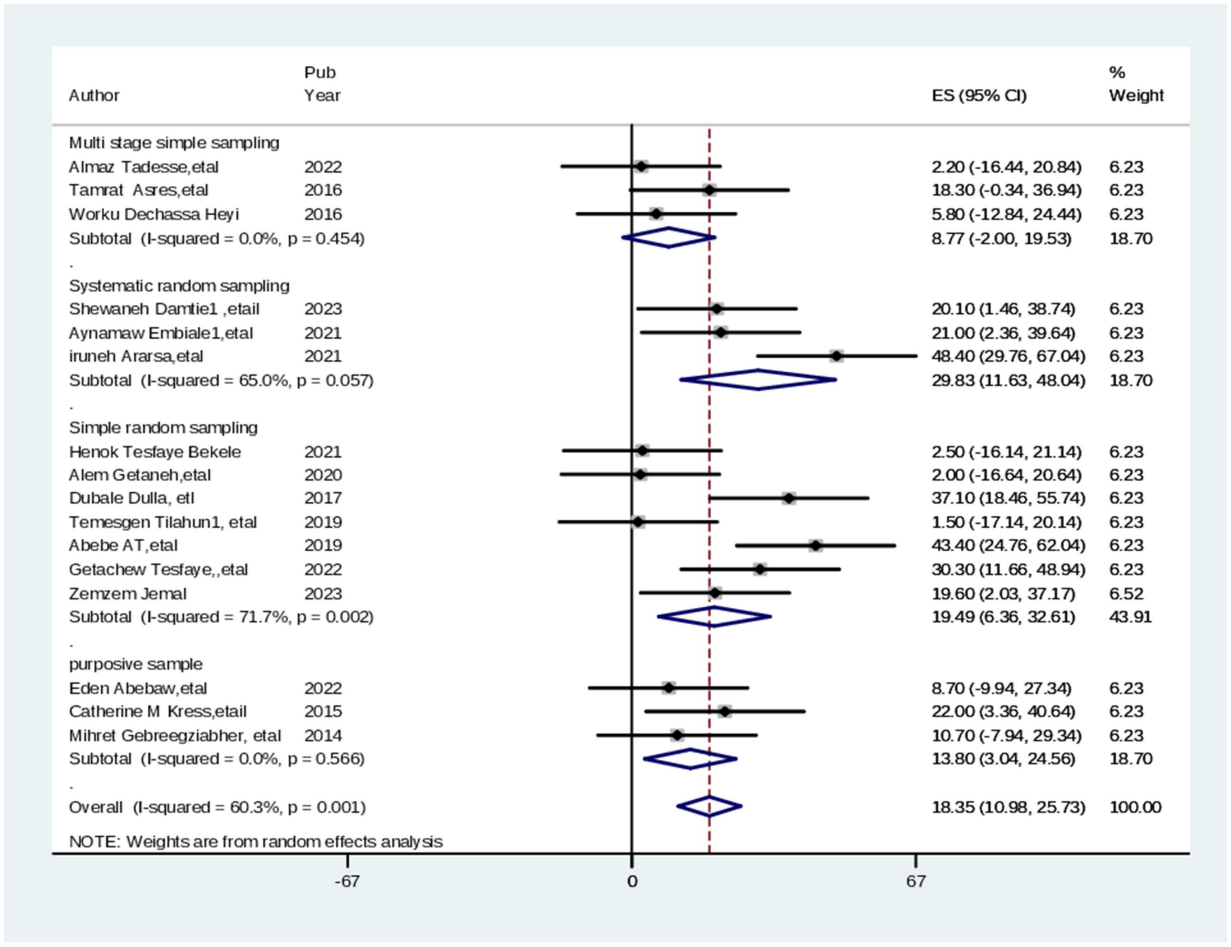
Subgroup analysis (by sampling technique) of studies included in meta-analysis on practice of cervical screening among healthcare professionals, 2024.

After stratification based on the study population, subgroup analysis was carried out to determine the sources of heterogeneity among the included articles. Therefore, subgroup analysis was conducted by sampling techniques for the level of satisfaction with maternal health services in Ethiopia. Based on the sampling technique categories the maximum level of satisfaction with maternal health services among parents was 74.63% (95% CI: 63.19, 86.06), I^2^ = 86.4, *p* = 000, in multistage random sampling method while 62.35% (95% CI: 55.41, 69.30), I^2^ = 75.5, *p* = 0.017, in simple random sampling Technique (Figure 3).

From systematic random sampling, the level of satisfaction with maternal health services among parents was 62.62% (95% CI: 51.59, 73.65), I^2^ = 84.6, *p* = 000. The level of heterogeneity is not identified. Hence, the effects of study participants, sample size, and year of study on the studies were examined using a meta-regression. However, there was no significant prediction of heterogeneity based on the assessed variables (sample sizes, study participants, and year of study) (Table 3). In the adjusted model, neither sampling sample size, study participants, nor the year of study indicated heterogeneity in the effect size was consistent with the pooled prevalence.

**Table 3:** Univariable meta-regression analysis results for the level of satisfaction with maternal health services among parents in Ethiopia.

Log odds	Coef. Std. Err.	*t*	P > t	[95% Conf. interval]
Participants	−0.1076045, 0.4682705	−0.23	0.827	−1.311332, 1.096123
Pub. year	0.0602613, 3.470183	0.02	0.987	−8.860129, 8.980652
Sample size	0.1074701, 0.468084	0.23	0.828	−1.095778, 1.310718
_cons	−121.7905, 7011.595	−0.02	0.987	−18145.67, 17902.09

### Sensitivity analysis

**Figure 4:**
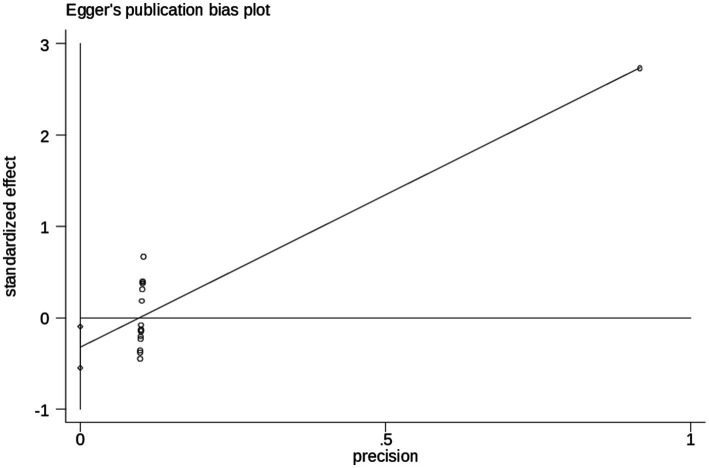
Egger’s test result for healthcare professionals and its associations with of cervical cancer screening practice in Ethiopia, 2024.

To evaluate the potential sources of heterogeneity on the pooled level of satisfaction with maternal health childhood vaccination services, a sensitivity analysis was performed using the random effects model. Hence, the sensitivity analysis revealed that the studies on the pooled satisfaction toward childhood vaccination services among parents (Figure 4).

Heterogeneity across studies might happen due to variations in study populations, such as age, sex, ethnicity, and health status, affecting outcomes. Differences in interventions, like level of education, duration, or delivery method, can also result in varied effectiveness. Variations in study sampling technique, such as sampling technique type versus systematic random sampling, introduce further heterogeneity, as each design has unique strengths and weaknesses. Statistical analysis methods can yield different effect size estimates, leading to inconsistencies.

### Publication bias

**Figure 5:**
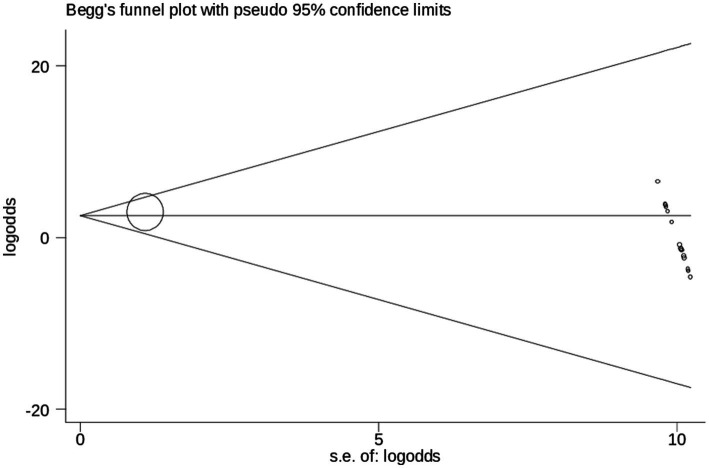
Begg’s test results for healthcare professionals and its association with of cervical cancer screening practice in Ethiopia, 2024.

According to objectively, Egger’s test result for publication bias showed statistically significant results (*p* = 0.005), which revealed that, there was evidence of absence of publication bias (*p*-value = 0.062) (Table 4), and the funnel plot results revealed symmetric shape, indicating the absence of publishing bias symmetrical observation of the funnel plot as demonstrated in (Figure 5).

**Table 4:** Egger’s test assessing publication bias.

Egger’s test					
Std_Eff	Coef.	Std. Err.	t	P > t	[95% Conf. Interval]
Slope	−0.6371019	0.0907085	−7.02	0.005	−0.8316523, −0.4425515
Bias	3.33423	0.0724992	45.99	0.062	3.178735, 3.489725

### Factors with parents’ level of satisfaction with maternal health services

**Figure 6:**
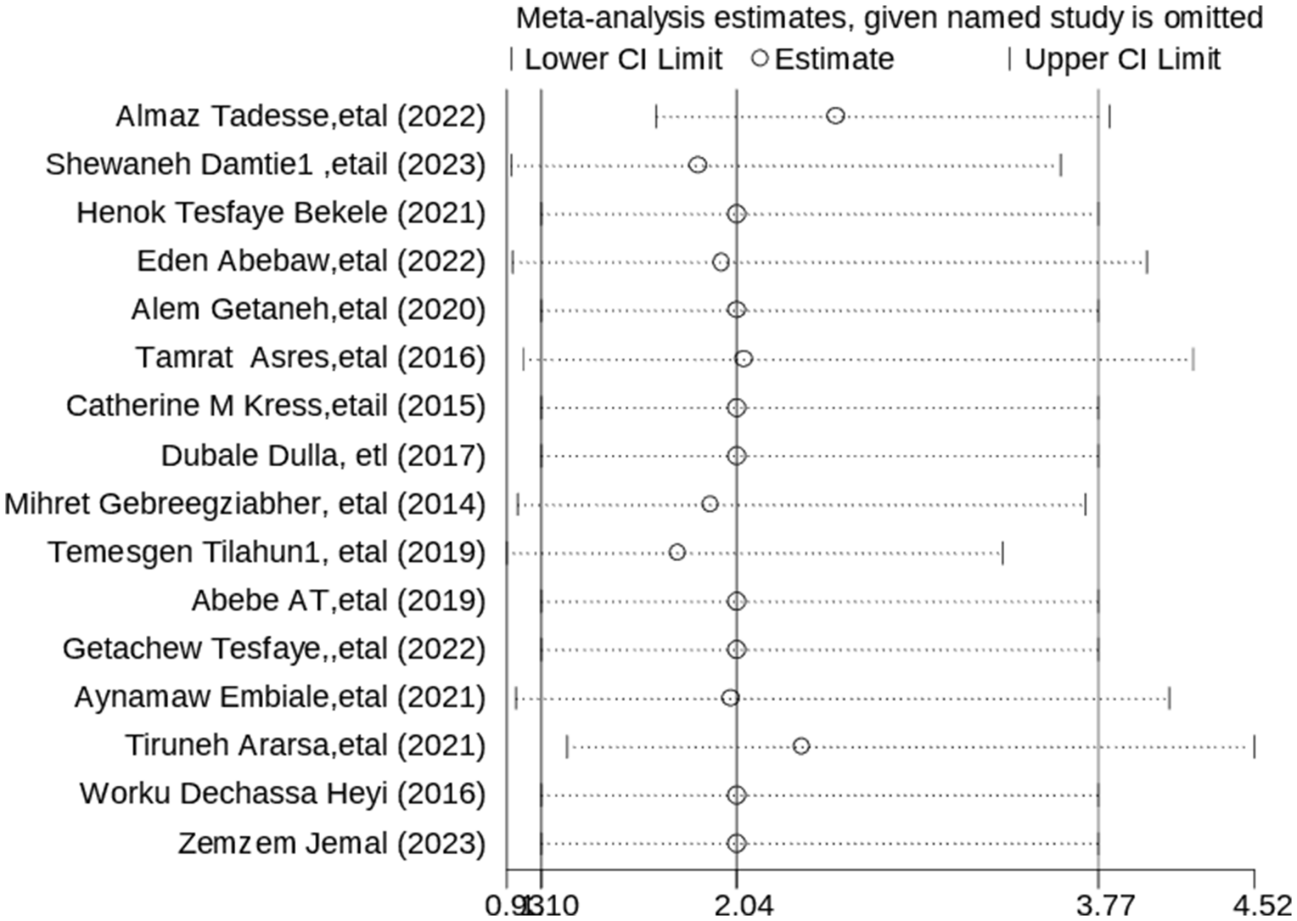
Output of sensitivity analysis of 16 studies.

**Figure 7:**
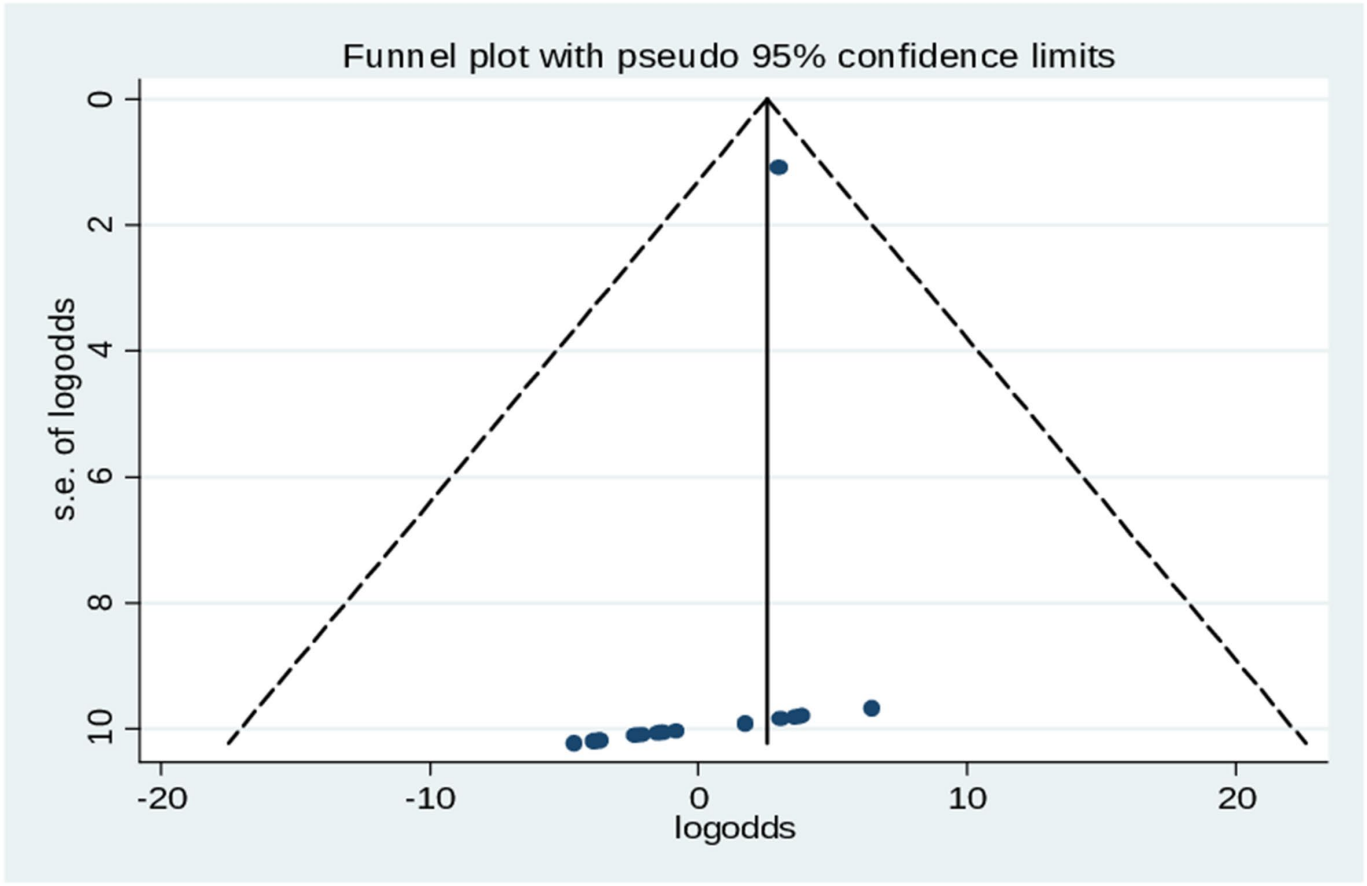
Funnel plot of the prevalence of cervical cancer screening practice in Ethiopia.

Associated factors contribute to the level of parents’ satisfaction with maternal health services. Based on this meta-analysis, different pooled variables relating to the level of satisfaction with maternal health services among parents were assessed. However, only four pooled variables were different among parents at the national level (Ethiopia). Thus, healthcare providers’ greeting or welcome approach, less waiting time, comprehensive knowledge, and attitudes were identified as significant determinants of the level of parents’ satisfaction with child vaccine services. The associations between the level of satisfaction with maternal health services and the knowledge of parents about childhood vaccination were computed from eight studies (29, 39–42). The odds of the level of the satisfaction with maternal health services were 1.93 times greater (AOR = 1.93; 95% CI: 1.09, 3.42) among parents who had good knowledge about maternal health compared with those who had poor knowledge (Figure 6). Arandom effect model analysis was used to reduce the heterogeneity: chi-squared = 19.35 (d.f. = 4), *p* = 0.001, and I^2^ = 79.3%. Four studies (3, 29, 39, 40) showed that parents who had a favorable attitude had a significant association with their level of satisfaction with maternal health during childhood. Thus, parents who had a favorable attitude toward maternal health services were 3.23 times (AOR = 3.23; 95% CI: 1.86, 5.62) more likely to have a high level of satisfaction with maternal health than those who had an unfavorable attitude. A random effect model was also used in this meta-analysis as the included studies were characterized by high heterogeneity chi-squared = 94.42% (d.f. = 7) (*p* = 0.000), and I^2^ = 92.6% (Figure 7).

**Figure 8:**
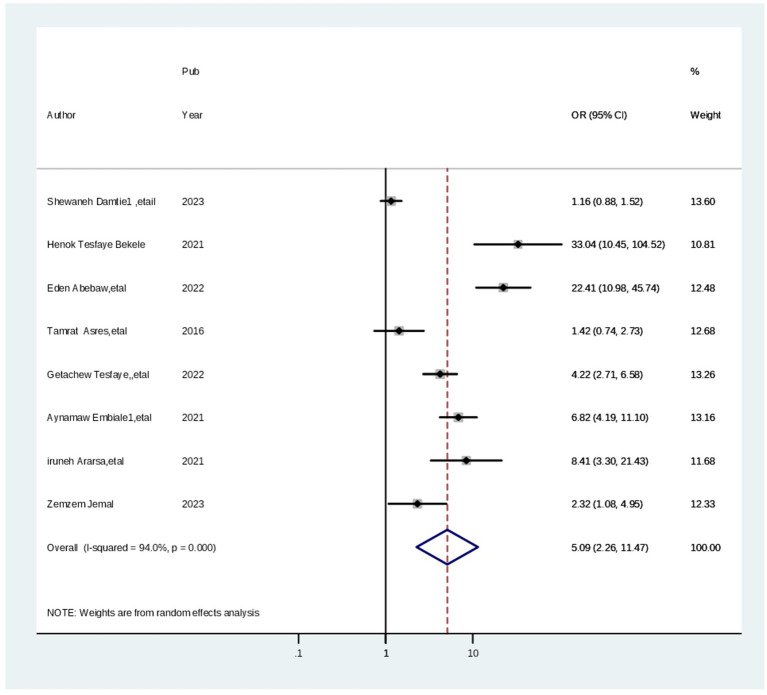
Forest plot showing the pooled odds ratio of the associations between having a knowledge and cervical screening practice among healthcare professionals in Ethiopia, 2024.

Moreover, the pooled odds ratio estimates level of satisfaction with maternal health in eight studies (3, 14, 29, 39, 40, 42–44) showed that parents who received greeting/welcome approach services from healthcare providers were 3.89 times (AOR = 3.89; 95% CI: 1.80, 8.42) more likely to have high level of satisfaction during child vaccination than parents who received disrespectful care. In this meta-analysis, the included studies were characterized by the presence of substantial heterogeneity (chi-squared = 153.62 (d.f. = 7), *p* = 0.000, and I^2^ = 95.4%). Thus, a random effect model analysis (Figure 8).

**Figure 9:**
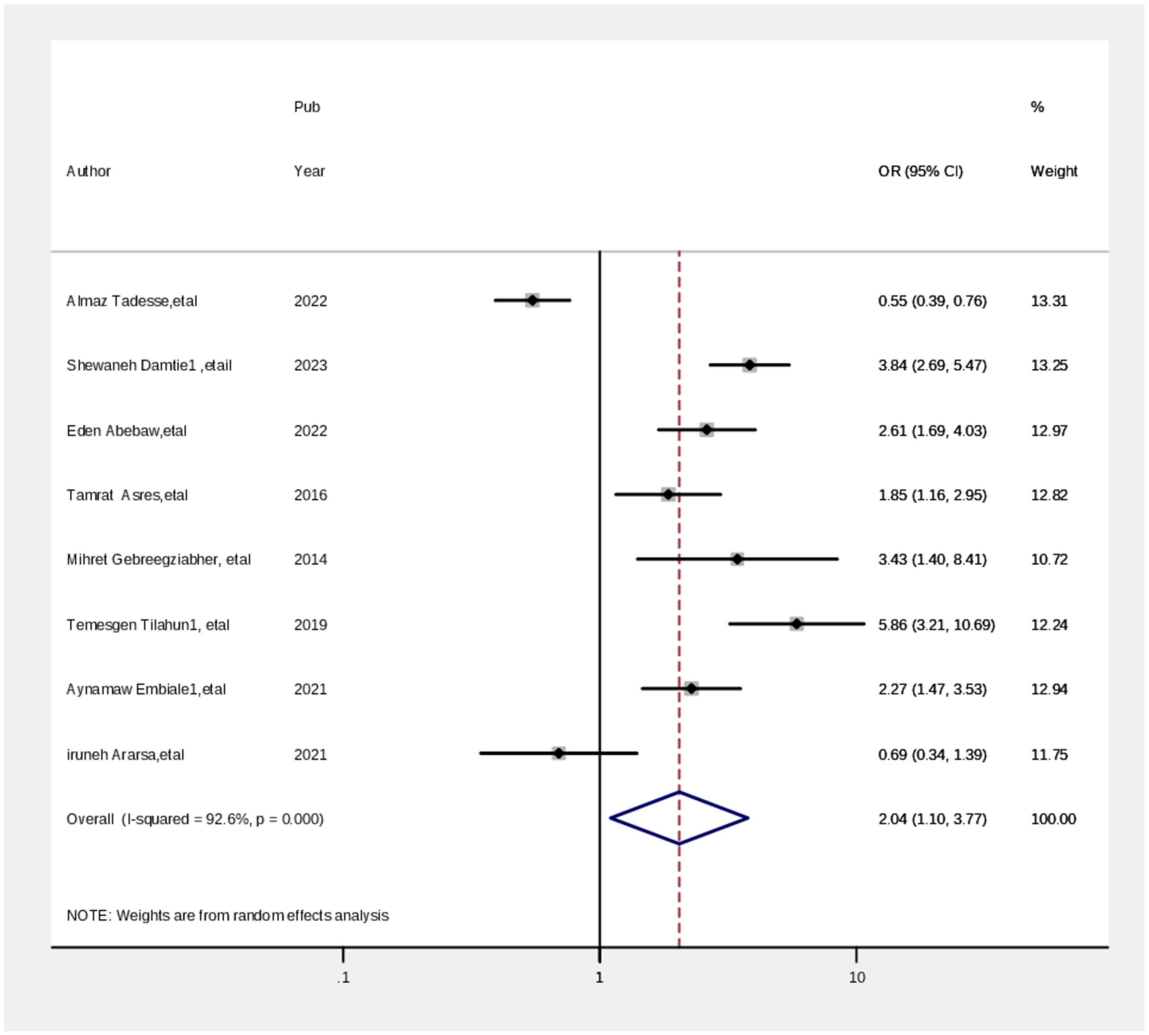
Forest plot showing the pooled odds ratio of the associations between having a welcoming approach among healthcare professionals in Ethiopia, 2024.

Finally, the pooled odds ratio estimates the level of parents satisfaction with child hood vaccination in eight (29, 33, 39–41, 45, 46) studies showed that parents who waited less than 30 min to receive maternal health services at health facilities were 2.59 times (AOR = 2.59; 95% CI: 1.25, 3.35) more likely to have high level of parents satisfaction during maternal health than parents who waited more time. In this meta-analysis, the included studies exhibited substantial heterogeneity (chi-squared = 98.58 (d.f. = 7), p = 0.000, and I^2^ = 92.9%). Thus, a random effect model analysis was carried out (Figure 9).

## Discussion

**Figure 10:**
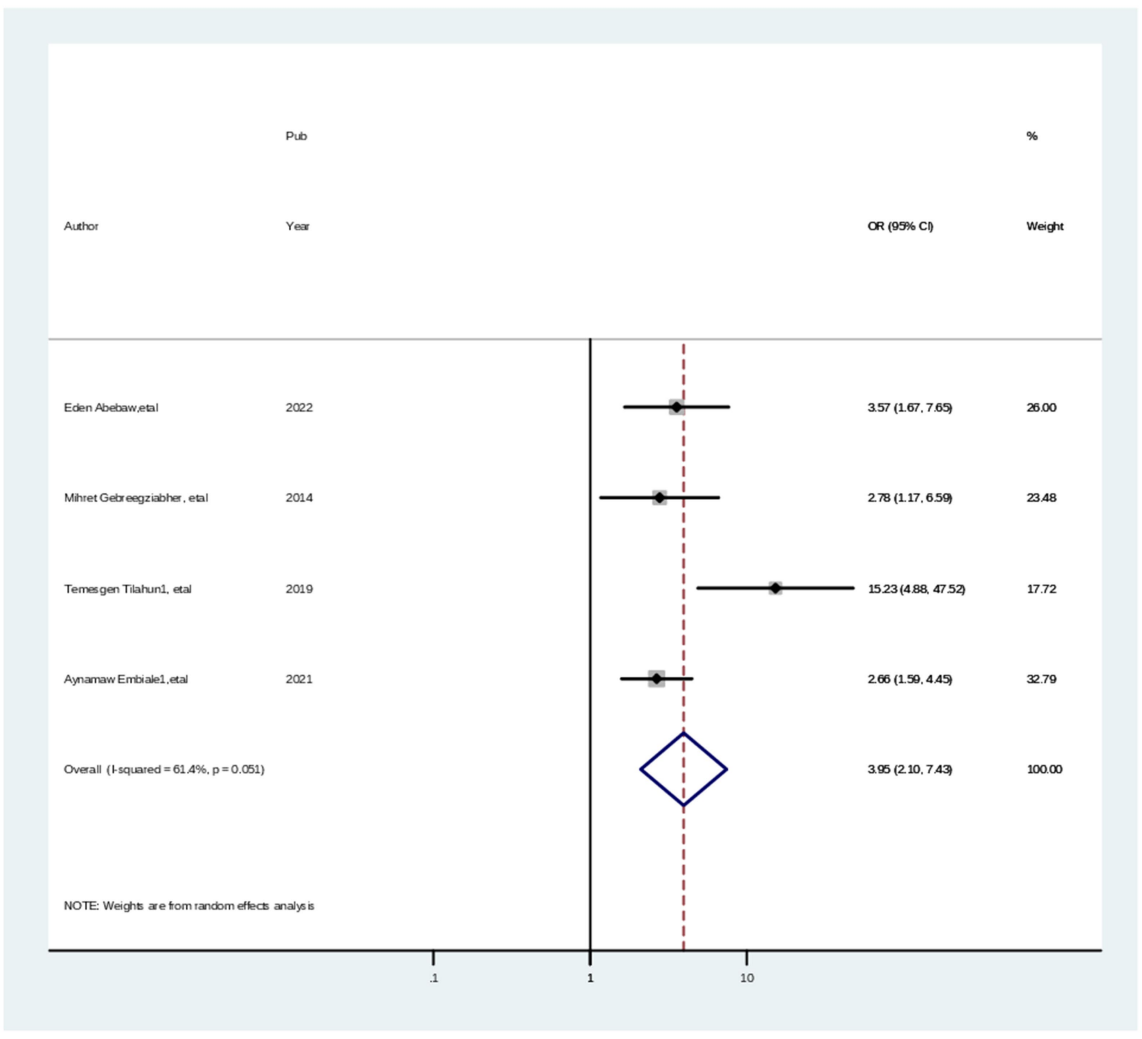
Forest plot showing the pooled odds ratio of the associations between took training cervical screening practice among the healthcare professionals in Ethiopia, 2024.

This systematic review and meta-analysis aimed to assess parental satisfaction with maternal health services and the associated factors in Ethiopia. Understanding parents’ satisfaction with childhood vaccine services in Ethiopia requires a comprehensive approach that considers the interplay of social, economic, and political factors. By addressing these factors, policymakers can improve vaccination services and increase overall satisfaction among parents, ultimately leading to higher vaccination rates and better public health outcomes (Figure 10).

The pooled level of parental satisfaction with maternal health services among parents was 65.6% (95% CI: 58.74, 72.44%). This indicates that nearly two-thirds of parents were satisfied with the maternal health services. This outcome has significant implications for the Ethiopian healthcare system. As outlined in the Ethiopian government’s Health Sector Transformation Plan II, improving the provision of equitable, high-quality, and comprehensive health services is central to the sector’s objectives, which extend beyond more health investments to promote universal coverage. The national agenda emphasizes the importance of quality of care. Therefore, the findings of this nationally representative study may provide valuable insights into the quality of maternal health healthcare services and support the implementation of the current national health sector plan (47).

The level of parental satisfaction with immunization in this study was significantly lower than the results reported in other countries, such as Egypt (95.2%) (48), India, (93.2%) (49), Saudi Arabia, (87.4%) (50), China, (90%) (51), Australia, (99.5%) (52), and Nigeria, (99.2%) (53). This large discrepancy may be attributed to differences in the quality of healthcare services and variations in the methods used to estimate parents’ satisfaction levels. In the Ethiopian primary study, parental satisfaction with maternal health services was assessed using the demarcation threshold formula, whereas other studies often utilized mean and median cutoff points for their measurements. In addition to this, the lower educational status of the study participants may also contribute to this difference in satisfaction levels. Our findings indicated that parents’ satisfaction varied based on the specific measurement items used. Parents who were most satisfied with the services they received emphasized that having friendly healthcare personnel was a key factor in their satisfaction. A study conducted in India found that the degree of friendliness exhibited by service providers increased client satisfaction by 33.6% (13), a finding also lower than that of a study conducted in China (39).

Furthermore, this may be the result of differences in cultural beliefs, and social norms can vary from one country to another in terms of quality and accessibility factors that can have a significant impact on access to healthcare and maternal health services, and consequently affect satisfaction with these services. It is also possible to justify this claim by pointing out that healthcare infrastructure can vary from one country to another in terms of quality and accessibility. The availability of well-equipped healthcare facilities, including vaccination centers, may be fully accessible in some parts of Nigeria and Australia.

However, this pooled level of parental satisfaction in this review aligned with findings from other studies, such as 60% in Vietnam (54) and 72.3% in Iraq (55), which may be because of similarities in the satisfaction measurement items used. Furthermore, this study revealed that knowledgeable parents were associated with a higher level of satisfaction with maternal health services.

Parents who possessed good knowledge about childhood vaccination services were more likely to report a high level of parental satisfaction compared to their counterparts. This finding was supported by previous studies (13, 52). This could be because parents with adequate knowledge about the importance of childhood vaccinations are more likely to develop a higher level of satisfaction with the services provided. A piece of evidence from India also revealed that a high level of satisfaction was closely linked to knowledge of child immunization service factors (13).

Participants with a favorable attitude were 3.23 times more likely to report a high level of satisfaction with maternal health compared to those who had an unfavorable attitude. This can be attributed to the fact that individuals with a positive attitude toward maternal health services are more likely to recognize the benefits of these services for those in need. Additionally, having a positive perspective on the advantages and disadvantages of childhood immunization may encourage parents and caregivers to engage in a friendly manner while receiving services. This finding was consistent with various studies conducted in Ethiopia and Saudi Arabia (56, 57). Overall, a positive attitude toward maternal health services not only enhances satisfaction but also fosters a more supportive and cooperative interaction between caregivers and healthcare providers.

Moreover, the results demonstrated that healthcare providers’ approaches were significant factors contributing to parental satisfaction with maternal health services. Specifically, the findings revealed that a welcoming or greeting approach by healthcare providers positively influenced parents’ satisfaction levels. For instance, parents who received greetings from care providers during vaccination services were 3.89 times more likely to express satisfaction compared to those who did not. Establishing effective and friendly interactions between healthcare providers and parents can help address the concerns of vaccine-supportive parents and encourage hesitant parents to accept vaccinations. This review was supported by studies conducted in Ghana, which indicated that respectful care and good communication between healthcare providers and parents were essential components of quality medical practice (19, 58). A possible justification that has the potential to be raised is that greetings can convey care, empathy, and support. For mothers who may be anxious or stressed about their child’s healthcare visit, receiving a friendly greeting can help alleviate those emotions. Feeling emotionally supported contributes to a positive service experience and improves satisfaction. A likely explanation is that when healthcare providers greet mothers warmly and respectfully, it fosters a positive rapport and creates a welcoming atmosphere. This personal touch improves the overall experience and contributes to increasing satisfaction with the service received. Therefore, implementing multidisciplinary interventions throughout the healthcare continuum to address dissatisfaction and provide training in respectful and compassionate care approaches is crucial for strengthening the overall quality of care.

Finally, this systematic review and meta-analysis found that a shorter service waiting time (less than 30 min) was a significant predictor of parental satisfaction with maternal health services. Parents who waited less than 30 min were 2.58 times more likely to report a high level of satisfaction with maternal health services compared to those who experienced longer wait times at health facilities. Similarly, reviews from China and Nigeria indicated that caregivers who waited longer than 30 min were less likely to be satisfied than those who had shorter wait times (53, 59). This similarity may be attributed to the subjective assessments and expectations of caregivers, as well as their acceptance of services under comparable circumstances. Some caregivers prioritize the services they desire, regardless of the length of time they must wait for those services. Quality assurance activities that assess the level of parental satisfaction during maternal health services can provide valuable insights into identifying children who are less likely to receive timely vaccination services.

### Strengths and limitations

To the best of our knowledge, all sections of the manuscript were written according to the PRISMA 2020 guidelines, and the quality of each study was assessed using the Newcastle-Ottawa Scale quality assessment tool. Although sensitivity analyses and subgroup analyses were employed to mitigate the influence of heterogeneity among the included studies remained significant. The nature of cross-sectional studies can identify associations between variables but cannot establish cause-and-effect relationships. Since data is collected at one point in time, it is difficult to determine whether one variable influences another. Although this comprehensive review does not represent all 14 regions of Ethiopia, it has been conducted in seven regions of the country. To be deeply understood, conducting qualitative and longitudinal studies with participants is recommended for future research.

## Conclusion and recommendation

This review indicated that only two-thirds of parents expressed satisfaction with maternal health services. The key factors influencing this level of satisfaction included the healthcare providers’ welcoming approach, waiting times of less than 30 min at health facilities, and the parents’ knowledge and attitudes toward vaccination.

To enhance parental satisfaction in Ethiopia and similar contexts, it is essential to address these identified factors. This includes improving the healthcare delivery system by ensuring timely and respectful maternal care, particularly through welcoming approaches. Additionally, it is recommended that the Ethiopian Ministry of Health implement respectful maternity care training for healthcare providers. This multifaceted approach could significantly improve the experience of parents and ultimately enhance satisfaction with healthcare services.

## Data Availability

The original contributions presented in the study are included in the article/Supplementary material, further inquiries can be directed to the corresponding author.
