# Supplementary material for: Satisfaction with maternal health services and associated factors among parents in Ethiopia: a systematic review and meta-analysis
Source: Front Public Health. 2025 Sep 24;13:1401498. doi: 10.3389/fpubh.2025.1401498 (PMC12504499; doi:10.3389/fpubh.2025.1401498)
Supplement: Supplementary file 2 [file Table_2.DOCX]

Table 1A: Search Strategy and Results in PubMed database for parents’ satisfaction with childhood vaccine services and Associated Factors in Ethiopia.

| Data base | Search | Query | Search results |
| --- | --- | --- | --- |
| PubMed | #1 | ((((Satisfaction with childhood vaccine services associated factors among parents in Ethiopia) OR (prevalence of satisfaction with childhood Immunization services and associated factors among parents in Ethiopia)) OR (level of satisfaction with childhood Immunization services and associated factors among parents)) OR (women)) OR (caregivers in Ethiopia primary studies) | 1,809,085 |
|  | #2 | Serch:((("patient satisfaction"[MeSH Terms] OR ("patient"[All Fields] AND "satisfaction"[All Fields]) OR "patient satisfaction"[All Fields] OR (("womans"[All Fields] OR "women"[MeSH Terms] OR "women"[All Fields] OR "woman"[All Fields] OR "women s"[All Fields] OR "womens"[All Fields]) AND ("personal satisfaction"[MeSH Terms] OR ("personal"[All Fields] AND "satisfaction"[All Fields]) OR "personal satisfaction"[All Fields] OR "satisfaction"[All Fields] OR "satisfactions"[All Fields] OR "satisfaction s"[All Fields])) OR (("parent s"[All Fields] OR "parentally"[All Fields] OR "parentals"[All Fields] OR "parented"[All Fields] OR "parenting"[MeSH Terms] OR "parenting"[All Fields] OR "parents"[MeSH Terms] OR "parents"[All Fields] OR "parent"[All Fields] OR "parental"[All Fields]) AND ("personal satisfaction"[MeSH Terms] OR ("personal"[All Fields] AND "satisfaction"[All Fields]) OR "personal satisfaction"[All Fields] OR "satisfaction"[All Fields] OR "satisfactions"[All Fields] OR "satisfaction s"[All Fields]))) AND (("childhood"[All Fields] OR "childhoods"[All Fields]) AND ("vaccin"[Supplementary Concept] OR "vaccin"[All Fields] OR "vaccination"[MeSH Terms] OR "vaccination"[All Fields] OR "vaccinable"[All Fields] OR "vaccinal"[All Fields] OR "vaccinate"[All Fields] OR "vaccinated"[All Fields] OR "vaccinates"[All Fields] OR "vaccinating"[All Fields] OR "vaccinations"[All Fields] OR "vaccination s"[All Fields] OR "vaccinator"[All Fields] OR "vaccinators"[All Fields] OR "vaccine s"[All Fields] OR "vaccined"[All Fields] OR "vaccines"[MeSH Terms] OR "vaccines"[All Fields] OR "vaccine"[All Fields] OR "vaccins"[All Fields]) AND ("service"[All Fields] OR "service s"[All Fields] OR "serviced"[All Fields] OR "services"[All Fields] OR "services s"[All Fields] OR "servicing"[All Fields])) AND (("associate"[All Fields] OR "associated"[All Fields] OR "associates"[All Fields] OR "associating"[All Fields] OR "association"[MeSH Terms] OR "association"[All Fields] OR "associations"[All Fields]) AND ("factor"[All Fields] OR "factor s"[All Fields] OR "factors"[All Fields])) AND (("factor"[All Fields] OR "factor s"[All Fields] OR "factors"[All Fields]) AND ("associate"[All Fields] OR "associated"[All Fields] OR "associates"[All Fields] OR "associating"[All Fields] OR "association"[MeSH Terms] OR "association"[All Fields] OR "associations"[All Fields])) AND ("analysis"[MeSH Subheading] OR "analysis"[All Fields] OR "determination"[All Fields] OR "determinant"[All Fields] OR "determinants"[All Fields] OR "determinate"[All Fields] OR "determinated"[All Fields] OR "determinates"[All Fields] OR "determinating"[All Fields] OR "determinations"[All Fields] OR "determine"[All Fields] OR "determined"[All Fields] OR "determines"[All Fields] OR "determining"[All Fields]) AND ("epidemiology"[MeSH Subheading] OR "epidemiology"[All Fields] OR "prevalence"[All Fields] OR "prevalence"[MeSH Terms] OR "prevalance"[All Fields] OR "prevalences"[All Fields] OR "prevalence s"[All Fields] OR "prevalent"[All Fields] OR "prevalently"[All Fields] OR "prevalents"[All Fields])) OR ("level"[All Fields] OR "levels"[All Fields])) AND ("ethiopia"[MeSH Terms] OR "ethiopia"[All Fields] OR "ethiopia s"[All Fields]) | 13234 |
|  | #3 | Search: **(((((((((((patient satisfaction) OR (women satisfaction)) OR (parents' satisfaction)) AND (childhood vaccine services)) AND (associated factors))) AND (factors associated)) AND (determinants))) AND (prevalence)) OR (level)) AND (Ethiopia)**Filters**: Observational Study** | 77 |
|  | #4 | (((((("epidemiology"[MeSH Subheading] OR "epidemiology"[All Fields] OR "prevalence"[All Fields] OR "prevalence"[MeSH Terms] OR "prevalance"[All Fields] OR "prevalences"[All Fields] OR "prevalence s"[All Fields] OR "prevalent"[All Fields] OR "prevalently"[All Fields] OR "prevalents"[All Fields] OR ("level"[All Fields] OR "levels"[All Fields])) AND (("womans"[All Fields] OR "women"[MeSH Terms] OR "women"[All Fields] OR "woman"[All Fields] OR "women s"[All Fields] OR "womens"[All Fields]) AND ("personal satisfaction"[MeSH Terms] OR ("personal"[All Fields] AND "satisfaction"[All Fields]) OR "personal satisfaction"[All Fields] OR "satisfaction"[All Fields] OR "satisfactions"[All Fields] OR "satisfaction s"[All Fields]))) OR (("parent s"[All Fields] OR "parentally"[All Fields] OR "parentals"[All Fields] OR "parented"[All Fields] OR "parenting"[MeSH Terms] OR "parenting"[All Fields] OR "parents"[MeSH Terms] OR "parents"[All Fields] OR "parent"[All Fields] OR "parental"[All Fields]) AND ("personal satisfaction"[MeSH Terms] OR ("personal"[All Fields] AND "satisfaction"[All Fields]) OR "personal satisfaction"[All Fields] OR "satisfaction"[All Fields] OR "satisfactions"[All Fields] OR "satisfaction s"[All Fields]))) AND (("childhood"[All Fields] OR "childhoods"[All Fields]) AND ("vaccin"[Supplementary Concept] OR "vaccin"[All Fields] OR "vaccination"[MeSH Terms] OR "vaccination"[All Fields] OR "vaccinable"[All Fields] OR "vaccinal"[All Fields] OR "vaccinate"[All Fields] OR "vaccinated"[All Fields] OR "vaccinates"[All Fields] OR "vaccinating"[All Fields] OR "vaccinations"[All Fields] OR "vaccination s"[All Fields] OR "vaccinator"[All Fields] OR "vaccinators"[All Fields] OR "vaccine s"[All Fields] OR "vaccined"[All Fields] OR "vaccines"[MeSH Terms] OR "vaccines"[All Fields] OR "vaccine"[All Fields] OR "vaccins"[All Fields]) AND ("service"[All Fields] OR "service s"[All Fields] OR "serviced"[All Fields] OR "services"[All Fields] OR "services s"[All Fields] OR "servicing"[All Fields])) AND (("associate"[All Fields] OR "associated"[All Fields] OR "associates"[All Fields] OR "associating"[All Fields] OR "association"[MeSH Terms] OR "association"[All Fields] OR "associations"[All Fields]) AND ("factor"[All Fields] OR "factor s"[All Fields] OR "factors"[All Fields]))) OR ("factor"[All Fields] OR "factor s"[All Fields] OR "factors"[All Fields])) AND ("ethiopia"[MeSH Terms] OR "ethiopia"[All Fields] OR "ethiopia s"[All Fields])) AND (observationalstudy[Filter]) | 160 |
|  | #5 | Search: **#1 AND #2 AND #3** | 42 |

***NB: Date of search from*** April 3, 2024 to 30, 2024
